# Physiological and genomic insights into the lifestyle of arsenite-oxidizing *Herminiimonas arsenitoxidans*

**DOI:** 10.1038/s41598-017-15164-4

**Published:** 2017-11-03

**Authors:** Hyeon-Woo Koh, Moonsuk Hur, Myung-Suk Kang, Youn-Bong Ku, Rohit Ghai, Soo-Je Park

**Affiliations:** 1 Department of Biology, Jeju National University, 102 Jejudaehak-ro, Jeju, 63243 Republic of Korea; 2 Microorganism Resources Division, National Institute of Biological Resources, 42 Hwangyeong-ro, Incheon, 22689 Republic of Korea; 3 Institute of Hydrobiology, Department of Aquatic Microbial Ecology, Biology Center CAS, Na Sadkach 7, České Budějovice, 370 05 Czech Republic

## Abstract

Arsenic, a representative toxic metalloid, is responsible for serious global health problems. Most organisms possess arsenic resistance strategies to mitigate this toxicity. Here, we reported a microorganism, strain AS8, from heavy metal/metalloid-contaminated soil that is able to oxidize arsenite, and investigated its physiological and genomic traits. Its cells were rod-shaped and Gram-negative, and formed small beige-pigmented colonies. 16S rRNA-based phylogenetic analysis indicated that the strain belongs to the genus *Herminiimonas* and is closely related to *Herminiimonas glaciei* UMB49^T^ (98.7% of 16S rRNA gene sequence similarity), *Herminiimonas arsenicoxydans* ULPAs1^T^ (98.4%), and *Herminiimonas saxobsidens* NS11^T^ (98.4%). Under chemolithoheterotrophic conditions, the strain utilized some organic acids and amino acids as carbon and/or nitrogen sources but not electron sources. Further, the strain grew as a sulfur oxidizer in a complex medium (trypticase soy agar). Unexpectedly, most carbohydrates failed to support its growth as sole carbon sources. Genome sequencing supported these observations, and very few ABC transporters capable of oligo/monosaccharide uptake were identified in the AS8 genome. The genome harbored genes required for the colonization, flagella biosynthesis, urea degradation, and heavy metal and antibiotic resistance. Based on these polyphasic and genomic analyses, we propose that the strain AS8 be named *Herminiimonas arsenitoxidans*.

## Introduction

The toxic metalloid arsenic is widely distributed in both natural and anthropogenic environments in two inorganic forms: arsenite [As(III)] and arsenate [As(V)]^1^. Particularly, arsenic contaminations result from an excessive use of pesticides, herbicides, and medicinal products^2^. Chronic arsenic exposure leads to several human diseases, including cancer, and cardiovascular and neurological disorders^3^. Compared with arsenate, arsenite is more bioavailable and toxic owing to its higher hydrophobicity and affinity for sulfhydryl groups present in many cellular proteins involved in critical oxidative functions^4^. Further, the auto-oxidation rate of arsenite is slower than that of iron under aerobic conditions^5^. To reduce the toxicity, a variety of methods such as precipitation, membrane filtration, and absorption using nano- or chemical-coated particles have been proposed^6–8^. However, most processes have economic and/or physical (handling) complications to achieve optimal capacity for arsenic removal, and additional methods are desired, e.g. biological transformations using microbes.

Since the discovery of the bacterial oxidation of arsenite to arsenate, both heterotrophic and chemoautotrophic oxidizing bacteria, displaying wide phylogenetic diversity, have been isolated from various ecosystems and characterized^1,9^. The oxidation of arsenite is mediated by an arsenite oxidase (Aio) that contains molydopterin^10^. Interestingly, a new gene encoding the arsenite oxidase (named as Arx) coupled to nitrate reduction was identified in the chemoautotrophic arsenite oxidizer, *Alkalilimnicola ehrlichii* ^11^. Recently, Zhang *et al*. reported an anaerobic autotrophic arsenite-oxidizing bacterium related to *Paracoccus*, isolated from arsenic-contaminated paddy soil^12^. Heavy metals are normally harmful to microbial cells even at low concentrations^13^ because of undesirable redox reactions leading to protein malfunction and/or oxidative stress^14–16^. As such, microbes utilize multiple strategies to counter the toxic effects of metals, including reduced uptake, efflux, extracellular or intracellular sequestration, or chemical modification^17^.

To determine the physiological and genomic traits associated with arsenic metabolism, we attempted to isolate a microorganism from a heavy metal-contaminated soil in Daegu (Republic of Korea). We successfully isolated an arsenite-oxidizing bacterium belonging to the genus *Herminiimonas*. The genus *Herminiimonas* comprises six different species, i.e., *Herminiimonas aquatilis* ^18^, *H*. *arsenicoxydans* ^19^, *H*. *contaminans* ^20^, *H*. *fonticola* ^21^, *H*. *glaciei* ^22^, and *H*. *saxobsidens* ^23^. These microbes have been isolated from diverse environments, e.g., drinking water^18^, bottled mineral water^21^, an industrial wastewater treatment plant contaminated with arsenic^19^, lichen-colonized rock^23^, and Greenland glacial ice at a depth of 3042 m^22^ and they have also been identified as contaminants in the biopharmaceutical industry^20^. At the time of writing, only two of these species, *H*. *arsenicoxydans* ^24^ and *Herminiimonas* sp. CN^25^, had been analyzed by genomic sequencing. *H*. *arsenicoxydans* was isolated as an arsenite oxidizer from an industrial sludge heavily contaminated with arsenic, and was the first *Herminiimonas* strain for which a complete genome sequence was available^24^. On the other hand, the incomplete genome of *Herminiimonas* sp. CN was assembled from metagenomic data and the microorganism was identified as an uncultivated toluene-degrading bacterium in a denitrifying microcosm^25^. No putative genes for arsenite oxidation were identified in the *Herminiimonas* sp. CN genome. However, an arsenic resistance operon (*arsRDACB*) was found. We herein report (1) the taxonomic characterization of the new isolate that we propose to call *H*. *arsenitoxidans* AS8; and (2) the genomic traits of the strain AS8 compared with other *Herminiimonas* genomes, in particular, the *H*. *arsenicoxydans* genome. Finally, based on the detailed phylogenetic and genomic studies, we show that *Herminiimonas* sp. CN has been misclassified and is not a *Herminiimonas*. This study provides insights into the metabolism and ecological roles of the AS8 isolate in the terrestrial environment, and serves as a basis for improving the current understanding of bacterial adaptation to metal-contaminated and/or anthropogenic soils.

## Results and Discussion

### Isolate characterization

**Figure 1:**
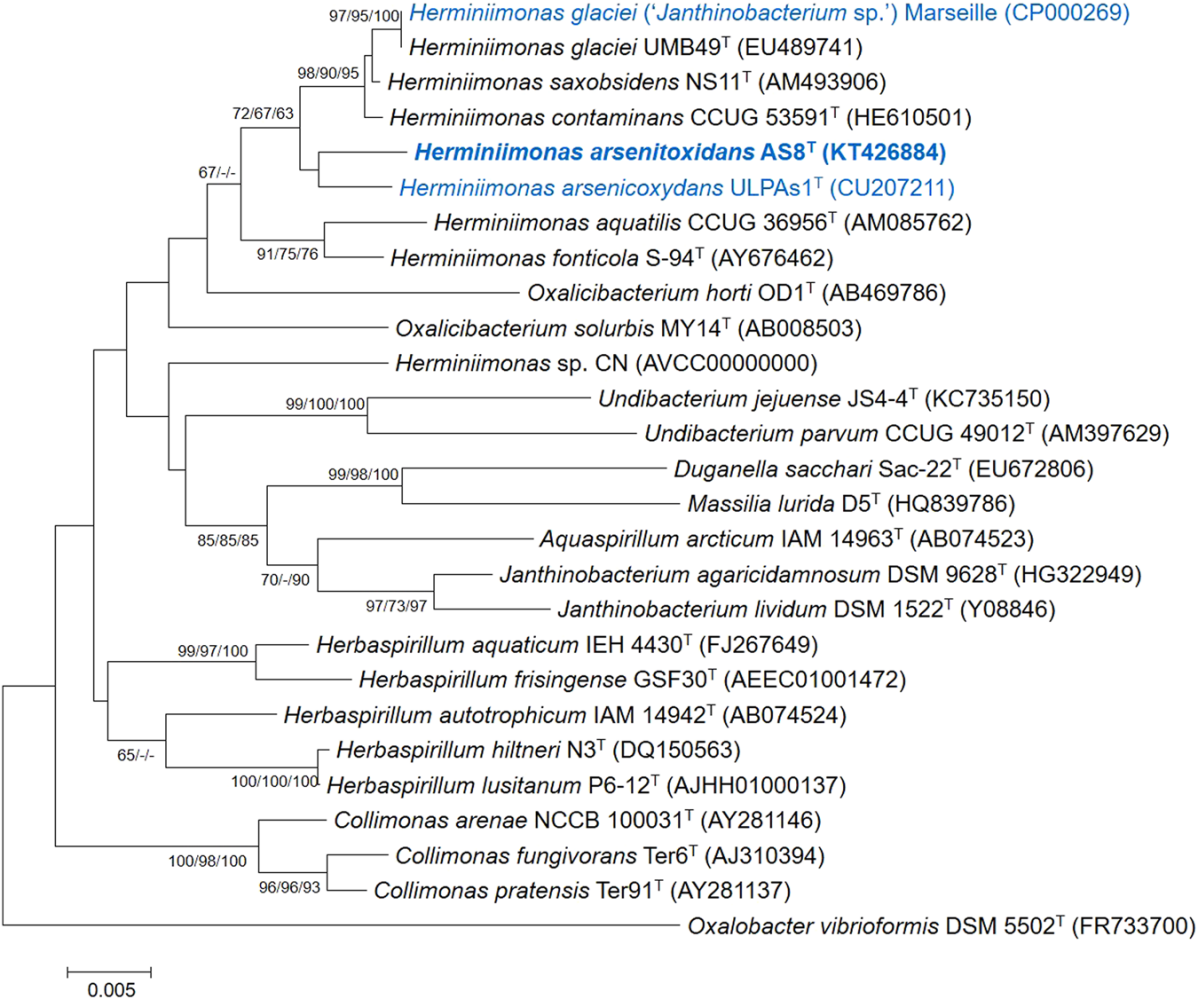
The phylogenetic tree of AS8 and *Herminiimonas* reference strains based on 16S rRNA gene sequences. The GenBank accession numbers are indicated in parentheses. *Oxalobacter vibrioformis* DSM 5502 ^T^ was used as an outlier. The tree was constructed using the neighbor-joining, maximum-likelihood, and maximum-parsimony methods. Bootstrap values are based on 1000 replicates; only bootstrap values above 60% are shown. Scale, 0.005 substitutions per nucleotide position. Color blue denotes the available complete genome sequences.

**Figure 2:**
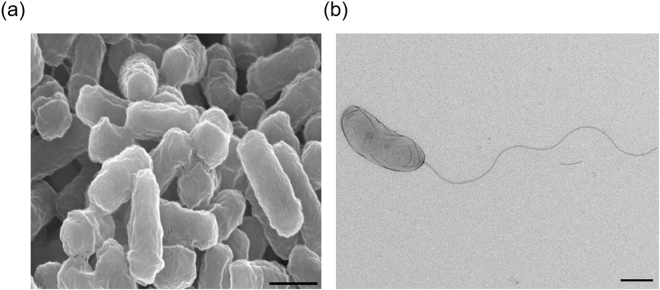
The cell morphology of strain AS8 as determined by scanning (**a**) and transmission (**b**) electron microscopy. Scale, 0.5 μm.

The strain AS8 was isolated from a heavy metal-contaminated soil near a drainage from an acid mine. The concentrations of heavy metals in the sample are shown in Supplementary Fig. S1. The phylogenetic analysis based on the 16S rRNA gene sequences indicated that AS8 was a member of the genus *Herminiimonas* (Fig. 1), as it shared high sequence similarity with *H*. *glaciei* UMB49^T^ (98.7%), *H*. *arsenicoxydans* ULPAs1^T^ (98.4%), and *H*. *saxobsidens* NS11^T^ (98.4%). The levels of DNA-DNA relatedness between the strain AS8 and *H*. *glaciei* UMB49^T^, *H*. *arsenicoxydans* ULPAs1^T^, and *H*. *saxobsidens* NS11^T^ were ca. 16.5%, 14.8%, and 14.8%, respectively. Based on these results, we concluded that the novel species may be a member of the genus *Herminiimonas*. The fatty acid profile of AS8 was consistent with that of the reference strains, as shown in Table 1, except that C_12:0_ 2-OH and C_18:0_ 3-OH were only present in AS8. Q-8 was the major ubiquinone. The polar lipids included diphosphatidylglycerol (DPG), phosphatidylethanolamine (PE), phosphatidylglycerol (PG), phosphatidylserine (PS), amino lipid (AL), and five unidentified lipids (ULs) (Supplementary Fig. S2). Furthermore, the AS8 cells were Gram-negative, oxidase- and catalase-positive, motile, aerobic, and rod-shaped, with a single polar flagellum (0.3–0.4 μm × 0.8–1.0 μm; Fig. 2). The colonies were beige-pigmented, circular, and smooth, with a diameter of 1.0–1.5 mm after 4 d of growth at 30 °C on R2A agar plates. The maximum growth rate (μmax) and the specific growth rate (μ) of the isolate were estimated as ca. 0.05 and 0.08, respectively, based on the optical density measurements at 600 nm (OD_600_) (Supplementary Fig. S3a). The tolerable and optimal growing conditions for AS8 were as follows: 20–40 °C (optimum: 25–30 °C), pH 3.0–9.0 (optimum: pH 5.0–6.0), and 0–1% (w/v) NaCl (optimum: 0.2–0.4% NaCl). In addition, the isolate oxidized thiosulfate (1 mM) and completely converted arsenite to arsenate (up to 10 mM, growth resistance up to 12 mM). However, the growth rate (μmax) tended to decrease with the increasing arsenite concentration (Supplementary Fig. S3b). Moreover, the strain tolerated high concentrations of arsenate (up to 80 mM; Supplementary Fig. S3c). Also, strain AS8 exhibited resistance to various other heavy metals, including Cu(II) (<5 mM), Pb(II) (<10 mM), Cd(II) (<10 mM), Co(II) (<10 mM), and Zn(II) (<10 mM). No growth was observed when arsenite was the sole energy source in the absence of growth factors, including yeast extract. Additionally, the biomass did not change under high-nutrient culture conditions (e.g., tryptic soy or nutrient agar). These results indicated that AS8 might experience energy stress under high-nutrient conditions or might have higher affinity for substrates in nutrient-limited environments. The physiological and biological characteristics of AS8 in comparison with *Herminiimonas* reference strains are summarized in Table 2.

**Table 1:** Cellular fatty acids of the novel strain and the closely related type strains from the genus *Herminiimonas*.

**Fatty acid**	***Herminiimonas arsenitoxidans*** **AS8**	***H***. ***saxobsidens*** **NS11** ^**T**^	***H***. ***glaciei*** **UMB49** ^**T**^	***H***. ***arsenicoxydans*** **ULPAs1** ^**T**^
C_12:0_	5.99	TR	—	—
C_14:0_	TR	TR	TR	4.92
C_16:0_	29.84	36.62	33.17	30.18
C_18:0_	TR	1.04	TR	1.23
C_10:0_ 3–OH	8.52	6.86	7.05	7.26
C_12:0_ 2–OH	3.40	—	—	—
C_18:0_ 3–OH	1.35	—	—	—
C_17:0_ cyclo	19.73	29.36	28.84	29.09
C_19:0_ cyclo ω8c	3.68	10.55	8.57	5.79
Summed Feature 3^†^	20.85	10.02	15.07	16.12
Summed Feature 7^‡^	—	—	—	1.55
Summed Feature 8^§^	5.36	3.00	4.86	2.83

All data are from this study. Fatty acids amounting to <1% of the total in all strains are not shown. —, not detected; TR, traces.

^†^Summed feature 3 comprises C_16:1_ *ω7c and/or* C_16:1_ *ω6c*.

^‡^Summed feature 7 comprises C_19:1_ *ω6c* and/or un18.846.

^§^Summed feature 8 comprises C_18:1_ *ω7c* and/or C_18:1_ *ω6c*.

**Table 2:** The differential characteristics of the novel strain AS8 and of *Herminiimonas* reference species.

**Characteristic**	***Herminiimonas arsenitoxidans*** **AS8**	***H***. ***saxobsidens*** **NS11** ^**T**^	***H***. ***glaciei*** **UMB49** ^**T**^	***H***. ***arsenicoxydans*** **ULPAs1** ^**T**^
Motility	+	+	+	+
**Cell size (μm):**				
Length	0.8–1.0	0.8	0.5–0.9	1.0–2.0
Width	0.3–0.4	0.4	0.3–0.4	0.5–0.7
Colony color	Cream	Cream	Brown	Yellow
**Growth temperature (°C)**				
Range	20–40	4–37	1–35	4–30
Optimum	25–30	ND	30	25
Optimum growth pH	5.0–6.0	7.0–7.5	ND	7.0–8.5
**Enzyme activity:** ^*^				
Alkaline phosphatase	+	−	−	+
Esterase (C4)	+	+	−	+
Esterase lipase (C8)	+	+	+	−
Lipase (C14)	−	+	+	+
Valine arylamidase	−	−	+	−
Crystine arylamidase	−	−	+	−
Trypsin	+	−	−	−
Acid phosphatase	+	−	+	−
Naphthol−AS−Bi−phosphohydrolase	−	−	+	−
*β*−Glucuronidase	+	−	−	+
*α*−Glucosidase	−	−	−	+
*β*−Glucosidase	+	−	−	−
*α*−Fucosidase	−	−	−	+
**Assimilation of:** ^*^				
*α*−Keto−glutaric acid	+	−	−	−
*α*−Hydroxy−butyric acid	−	−	−	+
Acetoacetic acid	−	−	−	+
Bromo−succinic acid	w	−	−	−
Citric acid	+	−	+	−
Formic acid	w	+	−	+
Glucuronamide	w	+	w	+
Methyl pyruvate	+	+	w	+
Propionic acid	+	+	−	+
d−Fructose−6−PO_4_	−	+	−	−
d−Malic acid	+	−	−	−
l−Alanine	+	−	−	+
l−Glutamic acid	+	−	+	−
l−Malic acid	+	+	+	−
DNA G+C content (%)	51.3	ND	59.0	54.3

All taxa were Gram-negative, and oxidase-and catalase-positive. All strains were positive for leucine arylamidase, l-lactic acid, *β*-hydroxy-d,l-butyric acid, and acetic acid. All strains were negative for production of urease and indole, *α*-chymotrypsin, *α*-galactosidase and *β*-glucosidase; hydrolysis of esculin ferric citrate and gelatin; fermentation of d-glucose; and utilization of *α*-d-lactose, *α*-d-glucose, *α*-keto-butyric acid, *β*-methyl-d-glucoside, *γ-*amino-butyric acid, *myo*-inositol, dextrin, gentiobiose, sucrose, stachyose, inosine, glycerol, gelatin, pectin, mucic acid, quinic acid, Tween 40, d-maltose, d-trehalose, d-cellobiose, d-turanose, d-raffinose, d-melibiose, d-salicin, d-mannose, d-fructose, d-galactose, d-fucose, d-sorbitol, d-mannitol, d-arabitol, d-glucose-6-PO_4_, d-aspartic acid, d-serine, d-galacturonic acid, d-gluconic acid, d-glucuronic acid, d-saccharic acid, d-lactic acid methyl ester, l-fucose, l-rhamnose, l-arginine, l-aspartic acid, l-histidine, l-pyroglutamic acid, l-serine, l-galactonic acid lactone, *N*-acetyl-d-glucosamine, *N*-acetyl-*β*-d-mannosamine, *N*-acetyl-d-galactosamine, *N*-acetyl neuraminic acid, 3-methyl glucose, glycyl-l-proline, and *p*-hydroxy-phenylacetic acid. + , positive; −, negative; w, weakly positive; ND, no data.

^*^Data for reference strains obtained in this study.

### General genomic features

Strain AS8 genome consisted of a single circular chromosome of ca. 3.78 Mbp (Supplementary Fig. S4) and with an overall GC content of 51.3%. The genome size was slightly larger, but with a lower GC content, than *H*. *arsenicoxydans*. In addition, the strain AS8 contained no extrachromosomal DNA, similarly to *H*. *arsenicoxydans* ^24^. Within the genome, two GC skew transitions corresponded to the *oriC* and terminus (Supplementary Fig. S4). The AS8 genome contained 3572 coding sequences (CDSs), comprising 89.8% of the genome, of which 20% encoded hypothetical proteins. The average gene length was 952 bp (316 amino acids), and 2743 (76.8%) predicted genes were assigned biological roles based on similarity analysis. The Cluster of Orthologous Groups (COG) classification categories were similar between the two genomes (strain AS8 and *H*. *arsenicoxydans*); additionally, the genes belonging to class R (general function prediction only) and S (function unknown) were numerous (Supplementary Fig. S4b). Interestingly, the genes in classes E, I, and P (for the transport and metabolism of amino acids, lipids, and inorganic ions, respectively), and K (transcription), were more numerous than in *H*. *arsenicoxydans*, suggesting that the AS8 strain is capable of utilizing a more diverse set of nutrients and may possess a survival advantage in contaminated environments^26^ (also see Table 2). Three rRNA operons were present in the chromosome, in addition to the encoded 46 tRNAs (Table 3).

**Table 3:** General features of the *H*. *arsenitoxidans* AS8, *H*. *arsenicoxydans*, and *H*. *glaciei* subsp. Marseille genomes.

**Characterization**	***Herminiimonas arsenitoxidans*** **AS8**	***H***. ***arsenicoxydans***	***H***. ***glaciei*** **subsp**. **Marseille**
Size (bp)	3,784,322	3,424,307	4,110,251
GC content (%)	51.3	54.3	54.2
Number of CDSs	3,572	3,228	3,819
Total CDS size (bp)	3,399,768	3,019,182	3,685,632
Coding percentage (%)	89.8	88.2	89.7
Average CDS length (NT/AA)	952/316	935/310	965/320
tRNAs	46	45	46
rRNA genes (operon)	6 (2)	6 (2)	6 (2)
Number of genes with assigned function	2743	2441	2887
Number of genes with assigned hypothetical protein	708	724	859
Number of predicted CDSs (pfam)	3103	2750	3246
Number of predicted CDSs (COG)	2431	2117	2460

All data are from this study with the exception of the genome size and GC content.

Eight putative genomic islands (GIs) were identified by the IslandViewer^27^, which integrates two prediction methods: SIGI-HMM (codon usage) and IslandPath-DIMOB (abnormal sequence composition) (Supplementary Table S1). The GIs comprised ca. 4% of the total genome; most GIs were similar to sequences in other taxonomic groups (e.g., alpha-, delta-, or gammaproteobacteria) and absent from the *H*. *arsenicoxydans* genome. The length of the GIs ranged from 4573 bp (GI 2, six genes) to 54,271 bp (GI 3, 58 genes). The GC content of the GIs ranged from 43.3% to 48.0%, which was lower than that of the whole genome (51.3%). The majority of the GI genes in the genome were related to cell wall biosynthesis, transposase (recombinase), or heavy metal and phage resistance, or they were genes of viral origin.

Unexpectedly, the strain was devoid of extrachromosomal DNA; most microorganisms isolated from contaminated and/or anthropogenic environments (e.g., containing heavy metals, hydrocarbons, or pesticides) harbor either a second chromosome or a plasmid to adapt to their habitat^28^. Moreover, only seven insertion sequences (ISs), belonging to ISL3, IS_ssgr_IS407, IS481, and IS21, were identified in the AS8 genome (Supplementary Table S2). Furthermore, the genome lacked the clustered regulatory interspaced short palindromic repeats (CRISPR) and CRISPR-related gene clusters. However, it contained a small number of transposons or transposon-related sequences (n = 5), in contrast to *H*. *arsenicoxydans* harbors 19 such sequences^24^. Interestingly, one prophage was detected in the AS8 genome, with 55 CDSs, including two integrases (Supplementary Table S3). Its length and GC content were approximately 38 kbp and 56.3%, respectively. Most genes in the prophage region were assigned hypothetical functions. Some CDSs were related to *Burkholderia* phages (e.g., BcepMu or phiE255). However, most genes showed no similarity to these phages. Two additional, but incomplete prophages were identified in the *H*. *arsenicoxydans* genome^24^.

### Central metabolism and respiration

The genomic analyses were consistent with the physiological characterization of the strain AS8 (Supplementary Table S5). For example, AS8 was able to metabolize only a limited number of organic substrates, such as lactate, glutamate, citrate, carbohydrates, and some amino acids, which corresponded with the transport systems identified in the genome. This was similar to *H*. *arsenicoxydans*, which uses similarly few substrates for growth^19,24^. Neither of the two strains (AS8 and *H*. *arsenicoxydans*) was a photoautotroph or chemolithoautotroph; no pathways for carbon fixation were found in their genomes. By contrast, many other species of the *Burkholderiales* order (*Betaproteobacteria*) are able to utilize various carbon and/or energy sources^29^. The AS8 genome also contained a complete carbohydrate metabolism pathway, including the glycolysis (the Embden-Meyerhof-Parnas pathway)/gluconeogenesis, pyruvate oxidation, tricarboxylic acid (TCA), pentose phosphate, and Entner-Doudoroff pathways. Interestingly, we also identified genes encoding the glyoxylate cycle, as an alternative cycle for acetate assimilation^30^. The genes involved in poly-3-hydroxybutyrate (PHB) biosynthesis or degradation were identified as well, i.e., *phbA*, *phbB*, *phbC*, and *phbZ*, which encoded an acetyl-CoA acetyltransferase, an acetoacetyl-CoA reductase, a polyhydroxyalkanoate synthase, and a poly-beta-hydroxyalkanoate depolymerase, respectively. The AS8 genome encoded two complete phosphorous uptake systems, i.e., the inorganic phosphate transport (Pit) and phosphate-specific (Pst) transport systems, as well as proteins related to phosphate storage as high-energy polyphosphate granules. These energy storage materials are considered to be inessential for survival, but they enhance the survival or resistance under several stress conditions^31,32^.

Arsenate reductase (ArsC) and the high-affinity and -activity phosphate uptake (Pts) operon allows arsenate to enter the cells via a phosphate transporter because arsenate is a structural analogue of phosphate^24,33^. Therefore, arsenic compounds have been suggested to affect the essential phosphate-related metabolism, such as oxidative phosphorylation, by replacing phosphate with arsenate^34^. The presence of a transposase and integrase upstream and downstream of these operons in the genome suggested that the Ars and Pts operons may have been acquired by horizontal transfer.

Additionally, the AS8 genome harbored genes necessary for the aerobic respiration, including those for complex I (NADH:ubiquinone oxidoreductase), complex II (succinate dehydrogenase), and complex III (cytochrome *bc*1 complex). Two types of terminal cytochrome *c* oxidase genes, which allow the oxygen to serve as a terminal electron acceptor, were found. One operates under high oxygen conditions as a cytochrome *c* oxidase *caa*3-type complex, and the other operates under low oxygen tension as a *bo*3- and *bd*-quinol oxidase^35^. However, genes involved in the production of the *cbb*3 cytochrome oxidase were identified. Normally, two main types of respiratory cytochrome oxidases are present in bacteria. Nevertheless, the AS8 genome harbored other multiple respiratory pathway genes, including sulfur compounds (e.g. thiosulfate). By contrast, no genes related to denitrification were observed, with the exception of a nitrite reductase. Therefore, strain AS8 is likely capable of growing under various oxygen concentrations and with various inorganic electron donors, such as reducing sulfur compounds.

**Figure 3:**
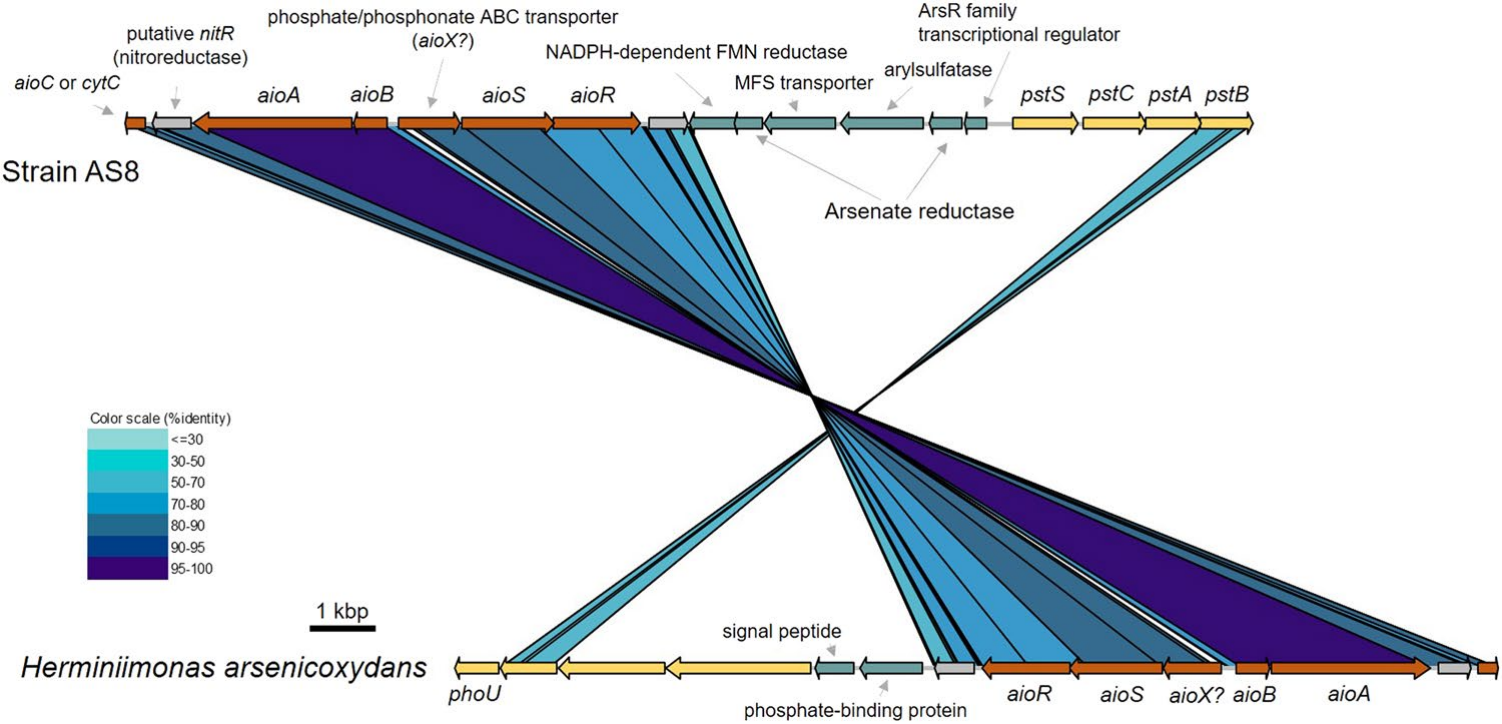
The comparison and organization of arsenite-related gene clusters identified in the AS8 and *H*. *arsenicoxydans* genomes. The homologous genes are connected by shaded regions, and the shading corresponds to percent identity, as determined by TBLASTX. The genes encoding hypothetical proteins are depicted in gray.

Further, we identified 37 genes coding for cytochromes. Among them, one gene (AS8-cds1561) was located downstream of the arsenite oxidase operon (*aioABC*). Moreover, a regulatory protein and a signal transduction protein involved in the regulation of *aioAB* (i.e., *aioS*) were found upstream of the *aio* operon. Therefore, it appears that the expression of these three genes is regulated in the presence of arsenite. Further, the gene order within the Aio operon was conserved, as in *H*. *arsenicoxydans* and in most *Proteobacteria* genomes^1^ (Fig. 3). However, neither *S*-adenosylmethionine methyltransferase (ArsM, arsenic methylation)^36^ nor the putative *arsM* genes were identified in the AS8 genome, although several genes were annotated as methyltransferase (i.e., *S*-adenosylmethionine-dependent methyltransferase, SAM-dependent methyltransferase). In addition, no genes related to the arsenic metabolism were identified in the genome of *H*. *glaciei* subsp. Marseille, with the exception of two copies of the *arsB* gene encoding the arsenite efflux transporter.

Sulfur metabolism in AS8 involves thiosulfate oxidation (SOX system) and assimilatory sulfate reduction (Cys operon). The genome also contained genes encoding taurine utilization proteins (*tauABCD*) and proteins involved in the mineralization of organic sulfonates (*ssuEADCB*) as sulfate or cysteine starvation-induced proteins^37^. In addition, two copies of a gene encoding cyanophycin synthetase were identified^38^. Cyanophycin is an insoluble amino acid polymer that is used as a nitrogen and carbon source by several cyanobacteria and some heterotrophic bacteria under stress conditions^39^. However, no cyanophycinase or cyanophycin protease gene was apparent in the AS8 genome^40^. Furthermore, the AS8 genome harbored 11 copies of the gene encoding rhodanese, an enzyme, which is important for cyanide detoxification because it converts cyanide to thiocyante^41^.

In contrast to *H*. *arsenicoxydans*, a complete gene set for urease (*ureABCDEF*) and its transporter (*urtABCDE*) with urea carboxylase was identified in the AS8 genome. Because terrestrial ecosystems are fertilized by urea, a dissolved nitrogen source, the capacity of the AS8 strain for urea utilization may confer a selective advantage in that niche. Moreover, the presence of putative dimethyl sulfoxide (DMSO) reductase genes (*dmsABC*) in the AS8 genome indicated that DMSO could act as a possible electron acceptor for anaerobic respiration.

### Motility, chemotaxis, colonization, and gene regulation

A complete gene set for flagellar biosynthesis (type IV) and flagellar proteins was found in the AS8 genome (Supplementary Table S6). The strain AS8 possessed several chemotaxis-like genes or gene clusters (Supplementary Table S6). One gene cluster encoded chemotaxis proteins related to motility (*cheAWRDBYZ*). In addition, genes encoding the methyl-accepting chemotaxis proteins (MCPs), aerotaxis receptor (low-abundance signal transducer, Aer), and high-abundance serine (Tsr) receptors were scattered throughout the genome. Although the response mechanism of Aer and MCP is different, these proteins are known as major oxygen sensors for aerotactic behavior^42^, suggesting that AS8 might have a competitive advantage in the presence of oxygen gradients in stratified environments (i.e., the oxic-anoxic interface). The genome contained genes for a 17 two-component signal transduction system (TCSs) composed of sensor histidine kinases and their response substrates, e.g., molecules involved in quorum sensing, capsule synthesis, and redox and phosphate starvation responses.

Similarly to *H*. *arsenicoxydans*, genes related to colonization, e.g., exopolysaccharide and mannose-sensitive hemagglutinin operons, were also identified, suggesting the induction of a thick capsule in harsh environments^43^ and the interaction between AS8 and other microflora in the habitat^24^, respectively. In addition, we identified genes involved in the biogenesis of pili (or fimbriae) and twitching motility proteins, with the *tad* (tight adherence) locus that plays important roles in bacterial conjugation, bacteriophage absorption, mobility, and biofilm formation by adherence to other bacteria and/or surfaces^44,45^.

### Heavy metal resistance

As mentioned above, strain AS8 has a displays resistance to some heavy metals. We attempted to identify the related genes in the AS8 genome to find the linkage to physiological characterization. We were able to identify genes encoding metal efflux and resistance operons related to heavy metals and metalloids, including arsenic, in the genome (e.g., a cation transporter for cobalt/zinc/cadmium, silver) (Supplementary Table S7). In addition, we found genes (*copABCD*) involved in copper resistance in the genome. For Pb(II) resistance, one gene [*pbrR*, a Pb(II) responsive regulator] belonging to the MerR family regulator was identified. Three copies of the chromate resistance operon (*chrAB*) were identified in the genome. The identification for these genes might be correlated to a survival strategy of the strain AS8 in a contaminated habitat. Nevertheless, with the exception of Cr(III), the level of resistance concentrations (up to 5 mM) to copper, cadmium, cobalt, and zinc were much higher than those for *H*. *arsenicoxydans* ^24^. However, the resistance level was significantly lower than for arsenite, which might imply that the strain AS8 possesses a specific physiological adaptation to arsenite-rich environments or metabolism. In fact, arsenite as a metalloid is known to cause oxidative stress-induced cytotoxicity^46–48^. We also identified two catalase-, three superoxide dismutase-, and seven peroxidase-encoding genes in the AS8 genome (Supplementary Table S7). Moreover, the genome encoded glutathione *S*-transferase, peroxiredoxin, alkyl hydroperoxide reductase, and hydroperoxide reductase. We also identified genes encoding one hemerythrin and two bacterioferritins that act against oxidative stress^49,50^.

We also found five copies of the tellurium resistance gene (t*erC*). Although tellurium has no known biological function, it can be incorporated into amino acids by fungi^51^. Bacteria can take up tellurite, reducing it to elemental tellurium, which then accumulates in the cells. This mechanism might be associated with the biofilm mode of growth^52^. Putative nickel transporter genes (*nikABCDER*) were also found in the AS8 genome; multiple copies of *nikBCE* but not *nikADR* were identified. Most bacteria possess a high-affinity molybdate uptake system (ModABCD); we identified genes encoding components of the ModABC system, a molybdate-dependent transcriptional regulator (ModE)^53^, and the ABC-type tungstate transport system (TupAB). Molybdate (molybdenum) is an essential cofactor (Moco, molybdopterin) that interacts with the active site of various key enzymes (oxomolybdenum enzymes), including nitrogenase and nitrate reductase as iron^54,55^. These enzymes play a critical role in anaerobic respiration. In fact, many anaerobic archaea and some bacteria appear to be molybdenum-independent, but require tungsten for growth^56^. Therefore, it might be possible that these uptake systems confer an ecological benefit (e.g. respiration) for increasing survival of the strain AS8 in anoxic conditions.

### Transporters and secretion systems

As mentioned above, the strain AS8 possesses genetic and phenotypic adaptations to metal-rich terrestrial environments in the form of multiple heavy metal efflux systems and transporters, some of which might have been derived from the neighboring microorganisms by horizontal transfer. These horizontally acquired sequence homologues were related to other genera of *Betaproteobacteria* and other proteobacterial classes, even *Euryarchaeota*. The AS8 genome harbored genes encoding transporters of amino acids (e.g., branched-chain and polar), organic acids, peptides, lipoproteins, lipooligosaccharides, and lipopolysaccharides. No gene sets for typical bacterial secretion systems, e.g., types I, II, and III, were identified in the genome.

The key genes of a putative glycine-betaine synthesis pathway (*betA* and *betB*, encoding choline dehydrogenase and betaine aldehyde dehydrogenase, respectively) were identified in the genome (Supplementary Table S8). The genes encoding ectoine biosynthesis proteins (EctABC), aspartate kinase (Ask), and mechanosensitive ion channel proteins (MscL and MscS) were also identified; these proteins enable the cell to export small proteins or compatible solutes in response to osmotic pressure changes within the cell^57–59^. Moreover, several osmoregulation receptors and transport systems encoded by the genome, such as the high-affinity potassium uptake system (Kdp) and sodium/proton antiporters (Nha), were found to respond to high salt stress^60^. Although the strain AS8 had a narrow salt tolerance range [growth on 0% to 1% (w/v) NaCl], it was resistant to hyperosmotic stress.

### Genome comparisons

BLASTP analysis of the AS8 proteins against the NCBI NR database consistently provided hits for *Janthinobacterium* sp. Marseille. Furthermore, we found that the 16S rRNA gene sequence of *Janthinobacterium* sp. Marseille was nearly identical to that of *H*. *glaciei* UMB49^T^ (99.9%), suggesting that this microbe is actually a *Herminiimonas* (Fig. 1). The microorganism was originally referred to as “*Minibacterium massiliensis*”, i.e., an ultramicrobacterium^61^. However, Loveland-Curtze *et al*.^22^ also noted that *Janthinobacterium* sp. Marseille is closely related to *H*. *glaciei* UMB49^T^, although there are some differences in the physiology of these strains (i.e., substrate utilization). Therefore, in the current study, we suggest and continue to refer to this organism as *Herminiimonas glaciei* subsp. Marseille.

**Figure 4:**
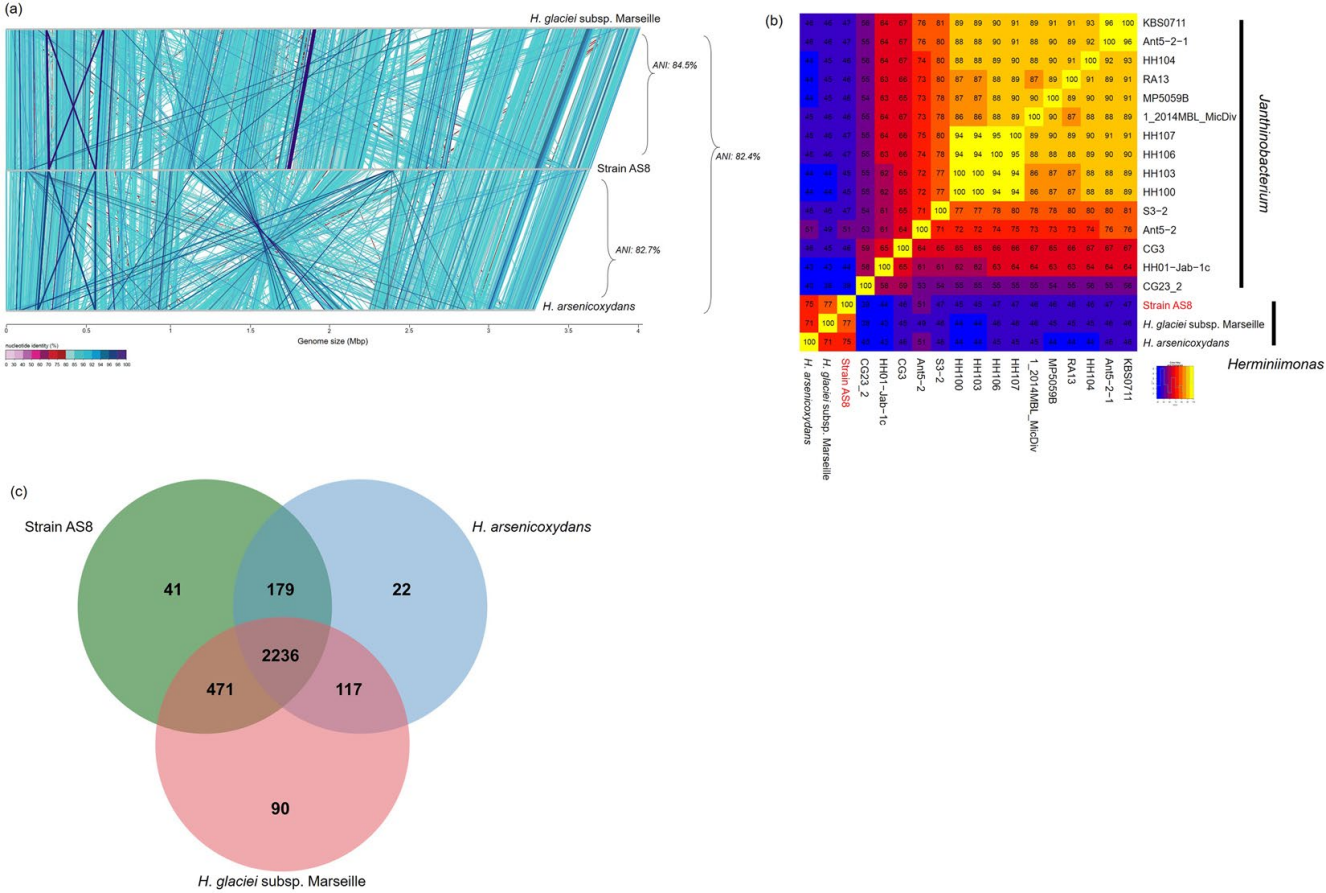
A comparative genome analysis of AS8, *H*. *arsenicoxydans*, and *H*. *glaciei* subsp. Marseille. (**a**) A whole-genome alignment of the three strains. The genomes have been linearized for simplicity and are arranged in the same order. The synteny of nucleotide sequences is indicated by vertical lines. (**b**) The matrix of percentages of conserved sequences calculated from the genomes for strain AS8, *Herminiimonas* spp., and *Janthinobacterium* spp. (**c**) Venn diagrams showing the numbers of CDSs shared between the predicted proteins of strain AS8 (green), *H*. *arsenicoxydans* (sky-blue), and *H*. *glaciei* subsp. Marseille (Red). The whole-genome orthologous gene comparisons were performed based on the OrthoVenn default value of OrthoVenn (http://probes.pw.usda.gov/OrthoVenn/start.php). The numbers indicate the total number of CDSs.

Consequently, in the current study, we used two additional genome sequences (*H*. *glaciei* subsp. Marseille and *H*. *arsenicoxydans* ULPAs1^T^) for genome comparisons. All the predicted CDSs indicated that AS8 was more closely related to *H*. *glaciei* subsp. Marseille (66.6% of total identified genes) than *H*. *arsenicoxydans* (14.3%). This agreed with the average nucleotide identity values (ANI, Fig. 4a). In particular, the ANI value between strain AS8 and *H*. *glaciei* subsp. Marseille (84.5%) was slightly higher than that between AS8 and *H*. *arsenicoxydans* (82.7%) (Fig. 4a). In addition, the mummer plots clearly indicated that the genome synteny between AS8 and *H*. *glaciei* subsp. Marseille was more pronounced than between AS8 and *H*. *arsenicoxydans* (Supplementary Fig. S5). This was supported by percentages of the conserved sequences (Fig. 4b)^62^. Best matches for ca. 210 CDSs (5.9%) originated within other clades, including unclassified groups, mainly, *Gammaproteobacteria*. This was consistent with the results of the orthologous cluster analysis^63^, as the AS8 genome (2236 CDSs) shared more genes with *H*. *glaciei* subsp. Marseille (i.e., 417) than *H*. *arsenicoxydans*, which shared ca. 120 genes with this genus (Fig. 4c). Only 41 AS8 clusters (104 CDSs) were considered as unique genes; the molecular functions of these genes were related to oxidoreductase, transporter, ligase, transferase, hydrolase, antioxidant, and peptidase activities, and to binding of ions, nucleotides, cofactors, and lipids (Supplementary Table S9). In addition, in the course of the orthologous cluster analysis, we identified 2236 CDSs as shared CDSs. It is possible that the strains (particularly, the *Herminiimonas* species) share or have conserved several genes related to metabolic properties via vertical gene transfer. These conserved (shared) genes might provide ecological advantages, including fitness and/or extended life strategy, to microorganisms in contaminated habitats^64^.

### Ecological strategies and interaction with other organisms

Interestingly, we also found two operons encoding the carbon monoxide (CO) dehydrogenase (CoxLMS); one operon was complete and related to the protein from *H*. *glaciei* subsp. Marseille, whereas the other, related to a that of *Herbaspirillum*, was missing one gene (e.g., CoxM). Microorganisms use CO as an energy source, via the CO oxidation pathway; this pathway (i.e., CO consumption) is an important process of the global carbon cycle, with an ancient metabolic evolutionary origin^65^. In addition, aerobic CO oxidation is known to occur in the soil; there are local biological sources (i.e. carbon dioxide) connected to the plant roots^66^.

The analysis of antibiotic susceptibility of AS8 revealed that the isolate was resistant to various antibiotics, including oxacillin (minimum inhibitory concentration, 5 μg), neomycin (30 μg), streptomycin (50 μg), penicillin G (10 IU per disc), gentamycin (10 μg), kanamycin (50 μg), sulfamethoxazole/trimethoprime (23.75/1.25 μg), clindamycin (10 μg), rifamycin (10 μg), lincomycin (10 μg), ampicillin (100 μg), erythromycin (10 μg), and vancomycin (10 μg), but not tetracycline (10 μg). Two types of β-lactamases (class A and class C enzymes) were encoded in the genome, explaining the ampicillin resistance. Moreover, we detected two genes predicted to encode homologues of the MacA and MacB proteins, which, along with the TolC outer membrane protein, form an ABC transporter system that exports macrolides. The MacA-MacB efflux pump promotes bacterial resistance to macrolides, e.g., azithromycin and erythromycin^67,68^. Additional genes encoding multidrug efflux systems for streptomycin, bicyclomycin/chloramphenicol, and vancomycin were also identified.

## Conclusions

The size of the *H*. *arsenitoxidans* AS8 genome is ca. 3.78 Mbp, with 51.3% GC content. As confirmed by physiological characterizations and supported by the genome analysis, AS8 is capable of surviving in a heavy metal-contaminated soil by detoxifying noxious compounds in its immediate environment. In addition, it harbored genes involved in toluene efflux (Ttg operon), but not degradation. The metabolic range of AS8 suggests that it is an obligate chemoheterotroph, although it also harbors some genes involved in inorganic compound metabolism, such as those of the thiosulfate oxidation system. However, as mentioned above, the growth of the strain under chemolithotrophic (i.e., oligotrophic) conditions with As(III) or thiosulfate as a sole energy and/or electron donor source was not observed, as the strain appeared to be a complete heterotroph and required organic compounds as an energy source. In the current study, we observed that the majority of AS8 genes involved in the detoxification and response to environmental stresses might have originated in other bacteria and have been acquired by horizontal gene transfer. Finally, the current study provides fundamental information for future studies, paving the way to a full understanding of the ecological niche of the genus *Herminiimonas* and its relatives in the contaminated ecosystems.

### Taxonomy

#### Etymology

The etymology of *Herminiimonas arsenitoxidans* sp. nov. is as follows: ar.se.nit.o’xi.dans (N.L. arsenitum, arsenite; N.L. part. Adj. oxidans, oxidizing; N.L. part. Adj. arsenitoxidans, oxidizing arsenite).

#### Locality

A heavy metal-contaminated site near an acid mine drainage located in Dalseong (Daegu Metropolitan City, Republic of Korea).

#### Characteristics

The cells are Gram-negative, aerobic, oxidase- and catalase-positive, motile, and rod-shaped (0.3–0.4 μm × 0.8–1.0 μm) after 4 d on an R2A agar plate. The colonies are cream-pigmented, circular, and smooth. The temperature for growth ranges between 20 and 40 °C; the growth pH ranges between 3.0 and 9.0. Optimal growth occurs at 25–30 °C and pH 5.0–6.0. The NaCl concentration allowing growth is 0–1% (w/v), with the optimum growth at 0.2–0.4% (w/v). Urease and indole are not produced. Gelatin and esculin are not hydrolyzed. The following constitutive enzyme activities are detected by API ZYM tests: alkaline phosphatase, esterase (C4), esterase lipase (C8), leucine arylamidase, trypsin, acid phosphatase, *β*-glucuronidase, and *β*-glucosidase; but the species is negative for lipase (C14), valine arylamidase, crystine arylamidase, *α*-chymotrypsin, naphtol-AS-Bl-phosphohydrolase, *α*-galactosidase, *β*-glucosidase, *α*-glucosidase, *N*-acetyl-*β*-glucosaminidase, *α*-mannosidase, and *α*-fucosidase. The following can be used as sole carbon and energy sources: methyl pyruvate, citric acid, *α*-keto-glutaric acid, d-malic acid, l-malic acid, propionic acid, l-arginine, l-glutamic acid, l-lactic acid, *β*-hydroxy-d,l-butyric acid, and acetic acid; and weakly positive for glucuronamide, bromo-succinic acid, and formic acid. The following are not used as sole carbon and energy sources: *α*-d-lactose, *α*-d-glucose, *α*-keto-butyric acid, *α*-hydroxy-butyric acid, *β*-methyl-d-glucoside, *γ*-amino-butyric acid, *myo*-inositol, dextrin, gentiobiose, sucrose, stachyose, inosine, glycerol, gelatin, pectin, mucic acid, quinic acid, Tween 40, acetoacetic acid, d-maltose, d-trehalose, d-cellobiose, d-turanose, d-raffinose, d-melibiose, d-salicin, d-mannose, d-fructose, d-galactose, d-fucose, d-sorbitol, d-mannitol, d-arabitol, d-glucose-6-PO_4_, d-fructose-6-PO_4_, d-aspartic acid, d-serine, d-galacturonic acid, d-gluconic acid, d-glucuronic acid, d-saccharic acid, d-lactic acid methyl ester, l-fucose, l-rhamnose, l-arginine, l-aspartic acid, l-histidine, l-pyroglutamic acid, l-serine, l-galactonic acid lactone, *N*-acetyl-d-glucosamine, *N*-acetyl-*β*-d-mannosamine, *N*-acetyl-d-galactosamine, *N*-acetyl neuraminic acid, 3-methyl glucose, glycyl-l-proline, and *p*-hydroxy-phenylacetic acid. Based on GEN III, antibiotic susceptibility was observed to troleandomysin, rifamycin SV, and lincomycin, whereas resistance were obtained for minocycline. The predominant quinone is ubiquinone Q-8, and the major fatty acids are C16:0 (29.84%), C17:0 cyclo (19.73%), and summed feature 3 (20.85%). The polar lipid profile of strain AS8 comprises diphosphatidylglycerol, phosphatidylethanolamine, phosphatidylglycerol, phosphatidylserine, amino lipid, and five unidentified lipids.

## Methods

### Site description and sample collection

Description for the isolation site and sampling collection are describe in detail in Supplementary Methods.

### Cultivation

The bacterial cultivation procedure and isolation medium used in this study are described in Supplementary Methods.

### Physiological and morphological characterization

Detailed procedures about the physiological and morphological characterization are described in Supplementary Methods.

### Phylogenetic analysis

The phylogenetic position and evolutionary distance were determined by 16S rRNA gene sequence. For detailed procedures, see Supplementary Methods.

### Chemotaxonomic analyses

Chemotaxonomic analyses for DNA hybridization, fatty acid composition, polar lipid, and quinone are described in detailed in Supplementary Methods.

### Genome sequencing, assembly, and annotation

Detailed information about genome sequencing, assembly, and annotation for the strain AS8 are described in Supplementary Methods.

### Data availability

The genome sequence of *H*. *arsenitoxidans* AS8 has been deposited in the ENA (European Nucleotide Archive) database under the accession number PRJEB17872.

## Electronic supplementary material

Supplementray Methods

Supplementary Figures

Supplementary Tables
