# Supplementary material for: Physiological and genomic insights into the lifestyle of arsenite-oxidizing Herminiimonas arsenitoxidans
Source: Sci Rep. 2017 Nov 3;7:15007. doi: 10.1038/s41598-017-15164-4 (PMC5670224; doi:10.1038/s41598-017-15164-4)
Supplement: Supplementary file 1 — Supplementray Methods [file 41598_2017_15164_MOESM1_ESM.doc]

**Physiological and genomic insights into the lifestyle of arsenite-oxidizing *Herminiimonas arsenitoxidans***

Hyeon-Woo Koh^1^, Moonsuk Hur^2^, Myung-Suk Kang^2^, Youn-Bong Ku^2^, Rohit Ghai^3^, & Soo-Je Park^1,*^

^1^Department of Biology, Jeju National University, 102 Jejudaehak-ro, Jeju 63243, Republic of Korea

^2^Microorganism Resources Division, National Institute of Biological Resources, 42 Hwangyeong-ro, Incheon 22689, Republic of Korea

^3^Institute of Hydrobiology, Department of Aquatic Microbial Ecology, Biology Center CAS, Na Sadkach 7, České Budějovice 370 05, Czech Republic

***Correspondence:**

Soo-Je Park

Tel.: +82-64-754-3524; Fax: +82-64-756-3541; E-mail: sjpark@jejunu.ac.kr

**Supplementary Information**

**SI Methods**

**Site description and sample collection.** The strain AS8 was isolated from a heavy metal (metalloid)-contaminated site near an acid mine drainage located in Dalseong (Daegu Metropolitan City, Republic of Korea; 35° 46' N 128° 40' E). The soil samples were collected from five sites (at 5 m intervals and 2–15 cm depth) in duplicate, transferred to sterile plastic tubes or bags, and stored at -80 °C for heavy metal analysis. Inductively coupled plasma atomic emission spectroscopy (ICP-OES; PerkinElmer Optima 7300 DV) was used to determine the soil concentrations of the various heavy metals. Before the analysis, the soil samples were sequentially filtered through 2.0 mm and 0.15 mm sieves, dried, and then digested with concentrated HNO_3_. The calibration was performed using a distilled water blank and standard solution. The concentrations of the selected heavy metals in each sample were determined in duplicate.

**Cultivation.** For the enrichment and isolation, a 1 g soil sample randomly selected from the contaminated soil samples was suspended in 10 mL of sterile 10-fold-diluted R2A broth medium (Difco, referred to as “DR2A” in the current study) or artificial freshwater medium (AFM, g/L: KCl, 1.3; KH_2_PO_4_, 0.2; NaCl, 23; NH_4_Cl, 0.5; CaCl_2_·2H_2_O, 0.1; MgCl_2_·6H_2_O, 3; and 1× vitamin solution^1^), vortex-mixed, and serially diluted; 100 μL aliquots were then used to inoculate R2A or AFM agar plates containing 3 mM NaAsO_2_ [As(III)]. The plates were then incubated at 30 °C for 2 weeks. Small single colonies capable of arsenite oxidation were transferred to new R2A agar plates and incubated again under the same conditions. One isolate, AS8, was cultured routinely on DR2A medium at 30 °C, and preserved as a suspension in R2A broth medium with glycerol (30%, v/v) at -80 °C. The concentrations of As(III) and As(V) were determined by colorimetry^2^.

**Physiological and morphological characterization.** The physiological and biochemical analyses were performed as previously described^3^. Gram-staining was performed with a BD Gram staining kit, according to the manufacturer’s instructions. The motility was tested by the hanging-drop method. Cell morphology and size were determined by scanning electron microscopy (Technai G2 Sprite twin; FEI) at the Korean Basic Science Institute, after negative staining with 1% (w/v) phosphotungstic acid. The anaerobic growth was tested using the BD GasPak EZ Anaerobe Container and GasPak EZ Anaerobe Pouch System over a period of 2 weeks. The oxidase and catalase activity tests were conducted using 1% (w/v) tetramethyl-*p*-phenylenediamine (Merck) and 3% (v/v) H_2_O_2_, respectively^4^. The growth at 5, 10, 20, 26, 30, 37, and 50 °C, and at pH 3.0–10.0 (at 0.5 pH unit intervals; pH was adjusted with 1 M NaOH and HCl), was assessed on R2A plates and/or in R2A broth after 1 week. For the pH experiments, four buffers were used (final concentration, 10 mM): Homo–PIPES (pH 4.5–5.0), MES (pH 5.0–6.5), Bis–Tris propane (pH 7.0–8.5), and CAPS (pH 9.0–10.0)^3^. The requirement for NaCl was tested by incubation for 1 week at 30 °C in R2A broth supplemented with NaCl (0–3%, w/v). The arsenite oxidation ability of strain AS8 was evaluated as described^2^. The arsenite-oxidizing (and resistance) test and arsenate resistance test were performed with 1–20 mM arsenite (1 mM intervals) and 1–100 mM arsenate (1, 5, 10–100, at 10 mM intervals), respectively, on R2A broth, with incubation for 1 week at 30 °C. Sulfur oxidation under aerobic conditions was determined at thiosulfate concentrations of 1–10 mM (3 mM intervals). The thiosulfate concentration was determined colorimetrically as previously described^5,6^. The heavy metal resistance tests were performed on R2A agar plates containing the following (1–10 mM): Cr(VI), Cu(II), Pb(II), Cd(II), Co(II), and Zn(II). The outcomes of all these tests, unless otherwise stated, were determined 2 weeks after the incubation, at OD_600_ (DeNovix DS-11D+ spectrophotometer, Willington DE). All the experimental materials containing heavy metals (including arsenic) were treated by a pollution treatment company to prevent secondary environmental pollution. Finally, the physiological characteristics were determined using the API 20NE, API ZYM (bioMérieux), and GEN III Microplate systems (Biolog Inc.), according to the manufacturers’ instructions.

**Phylogenetic analysis.** Extraction of the genomic DNA (gDNA) was performed using a commercial gDNA extraction kit (Geneall, Republic of Korea). The 16S rRNA gene was amplified from the chromosomal DNA by PCR using the universal bacterial primer set 27F (5′-AGAGTTTGATCMTGGCTCAG-3′) and 1492R (5′-TACGGYTACCTTGTTACGACTT-3′)^7,8^. The cycling conditions were as follows: 95 °C for 7 min; followed by 35 cycles of 95 °C for 0.5 min, 55 °C for 0.5 min, and 72 °C for 1.5 min; and a final extension of 72 °C for 7 min. The PCR products were purified using a PCR purification kit (Cosmo Genetech, Republic of Korea) and sequenced by Macrogen (Republic of Korea). The complete gene sequence was compiled using SeqMan software (DNASTAR). The 16S rRNA gene sequences of the related taxa were obtained from the GenBank database (http://www.ncbi.nlm.nih.gov/) and EzTaxon server^9^. Multiple alignments were performed using CLUSTALX^10^. The gaps were edited in BioEdit^11^. The evolutionary distance was calculated using the Kimura two-parameter model^12^. The phylogenetic trees were constructed using the neighbor-joining method^13^, maximum-parsimony method^14^, and maximum-likelihood method^15^ in MEGA7^16^ with bootstrap values based on 1000 replications^17^. In the current study, the reference strains selected for comparison were obtained from the German Collection of Microorganisms and Cell Cultures (DSMZ): *H. glaciei* UMB49^T^, *H. arsenicoxydans* ULPAs1^T^, and *H. saxobsidens* NS11^T^. Unless otherwise stated, all reference strains were grown on R2A broth under optimal culture conditions^18-20^.

**Chemotaxonomic analyses*.*** DNA hybridization was conducted fluorometrically in microwells as described by Ezaki et al.^21^ using photobiotin-labeled DNA from the isolated strain as a probe. To analyze fatty acid composition, AS8, *H.* *glaciei* UMB49^T^, *H.* *arsenicoxydans* ULPAs1^T^, and *H.* *saxobsidens* NS11^T^ were grown on R2A agar plates at 30 °C for 5 d to isolate the fatty acid methyl esters according to the protocol published in the Sherlock Microbial Identification System (MIDI, http://www.microbialid.com/PDF/TechNote_101.pdf). The fatty acid profiles were then determined by gas chromatography using the 6890N and 7683 autosamplers (Agilent Technologies), according to the manufacturer’s instructions, and the MIDI/Hewlett Packard Microbial Identification System^22^. The polar lipids were extracted from freeze-dried cells (100 mg) and analyzed as previously described^23^.

The quinones were extracted with a chloroform/methanol mixture (2:1, v/v), evaporated under vacuum, and re-extracted three times with *n*-hexane/water (1:1, v/v). Then, the crude quinone extract was concentrated and applied to a Sep-Pak Plus silica column (Waters). The quinone components were separated and identified by reversed-phase HPLC and photodiode array detection with internal and external standard quinones, as described^24^.

**Genome sequencing, assembly, and annotation*.*** gDNA was prepared using the FastDNA SPIN kit (MP Biomedical) according to the manufacturer’s instructions. The genome was sequenced in one SMRT cell on a PacBio RS II sequencing instrument (20 kb library, 320-fold coverage). The *de novo* genome assembly was performed using PacBio SMRT analysis software (version 2.3) and the HGAP2 hierarchical genome assembly process with default settings. After the assembly, the putative CDSs were predicted using Prodigal^25^. Protein sequences were annotated using the best BLAST hit against the NCBI NR database, and tRNAs were identified using tRNAscan-SE^26^. The similarity analysis was performed with COGs, TIGRfam, Pfam, and Kyoto Encyclopedia of Genes and Genomes database references, as described^27,28^. The GIs and repeat sequences were detected using the IslandViewer^29^ and Tandem Repeats Finder^30^, respectively. Orthologous gene clusters and prophage sequences for the isolate and reference genomes were identified using OrthoVenn and PHAST, respectively^31,32^. Duplicate genes in each genome were removed from the genomes before orthologous cluster analysis.

**SI References**

1 Park, B. J. *et al.* Cultivation of autotrophic ammonia-oxidizing archaea from marine sediments in coculture with sulfur-oxidizing bacteria. *Appl Environ Microbiol* **76**, 7575-7587, doi:10.1128/AEM.01478-10 (2010).

2 Simeonova, D. D. *et al.* Microplate screening assay for the detection of arsenite-oxidizing and arsenate-reducing bacteria. *FEMS Microbiol Lett* **237**, 249-253, doi:10.1016/j.femsle.2004.06.040 (2004).

3 Koh, H. W. *et al.* *Rhodanobacter aciditrophus* sp. nov., an acidophilic bacterium isolated from mine wastewater. *Int J Syst Evol Microbiol* **65**, 4574-4579, doi:10.1099/ijsem.0.000614 (2015).

4 Smibert, R. & Krieg, N. Phenotypic characterization: In Methods for General and Molecular Bacteriology P. *WashingtonDC: American Society for Microbiology* (1994).

5 Park, S. J. *et al.* *Thioalbus denitrificans* gen. nov., sp. nov., a chemolithoautotrophic sulfur-oxidizing gammaproteobacterium, isolated from marine sediment. *Int J Syst Evol Microbiol* **61**, 2045-2051, doi:10.1099/ijs.0.024844-0 (2011).

6 Voroteliak, V., Cowley, D. M. & Florin, T. H. Improved colorimetric determination of urinary thiosulfate to study intermediate sulfur metabolism in humans. *Clin Chem* **39**, 2533-2534 (1993).

7 Lane, D. in *Nucleic acid techniques in bacterial systematics* (eds E. Stackebrandt & M. Goodfellow) 115-175 (John Wiley & Sons, 1991).

8 Weisburg, W. G., Barns, S. M., Pelletier, D. A. & Lane, D. J. 16S ribosomal DNA amplification for phylogenetic study. *J Bacteriol* **173**, 697-703 (1991).

9 Kim, O. S. *et al.* Introducing EzTaxon-e: a prokaryotic 16S rRNA gene sequence database with phylotypes that represent uncultured species. *Int J Syst Evol Microbiol* **62**, 716-721, doi:10.1099/ijs.0.038075-0 (2012).

10 Thompson, J. D., Gibson, T. J., Plewniak, F., Jeanmougin, F. & Higgins, D. G. The CLUSTAL_X windows interface: flexible strategies for multiple sequence alignment aided by quality analysis tools. *Nucleic Acids Res* **25**, 4876-4882 (1997).

11 Hall, T. A. BioEdit: a user-friendly biological sequence alignment editor and analysis program for Windows 95/98/NT. *Nucleic Acids Symp Ser* **41**, 95-98 (1999).

12 Kimura, M. (Cambridge University Press, Cambridge, 1983).

13 Saitou, N. & Nei, M. The neighbor-joining method: a new method for reconstructing phylogenetic trees. *Mol Biol Evol* **4**, 406-425 (1987).

14 Fitch, W. M. Toward defining the course of evolution: minimum change for a specific tree topology. *Syst Biol.* **20**, 406-416 (1971).

15 Felsenstein, J. Evolutionary trees from DNA sequences: a maximum likelihood approach. *J Mol Evol* **17**, 368-376 (1981).

16 Kumar, S., Stecher, G. & Tamura, K. MEGA7: Molecular Evolutionary Genetics Analysis Version 7.0 for Bigger Datasets. *Mol Biol Evol* **33**, 1870-1874, doi:10.1093/molbev/msw054 (2016).

17 Felsenstein, J. Confidence limits on phylogenies: an approach using the bootstrap. *Evolution*, 783-791 (1985).

18 Loveland-Curtze, J., Miteva, V. I. & Brenchley, J. E. *Herminiimonas glaciei* sp. nov., a novel ultramicrobacterium from 3042 m deep Greenland glacial ice. *Int J Syst Evol Microbiol* **59**, 1272-1277, doi:10.1099/ijs.0.001685-0 (2009).

19 Muller, D. *et al.* *Herminiimonas arsenicoxydans* sp. nov., a metalloresistant bacterium. *Int J Syst Evol Microbiol* **56**, 1765-1769, doi:10.1099/ijs.0.64308-0 (2006).

20 Lang, E. *et al.* *Herminiimonas saxobsidens* sp. nov., isolated from a lichen-colonized rock. *Int J Syst Evol Microbiol* **57**, 2618-2622, doi:10.1099/ijs.0.65163-0 (2007).

21 Ezaki, T., Hashimoto, Y. & Yabuuchi, E. Fluorometric deoxyribonucleic acid-deoxyribonucleic acid hybridization in microdilution wells as an alternative to membrane filter hybridization in which radioisotopes are used to determine genetic relatedness among bacterial strains. *Int J Syst Evol Microbiol* **39**, 224-229 (1989).

22 Sasser, M. Identification of bacteria by gas chromatography of cellular fatty acids. (1990).

23 Komagata, K. & Suzuki, K.-I. Lipid and cell-wall analysis in bacterial systematics. *Method Microbiol* **19**, 161-207 (1987).

24 Hiraishi, A., Ueda, Y., Ishihara, J. & Mori, T. Comparative lipoquinone analysis of influent sewage and activated sludge by high-performance liquid chromatography and photodiode array detection. *J Gen Appl Microbiol* **42**, 457-469 (1996).

25 Hyatt, D. *et al.* Prodigal: prokaryotic gene recognition and translation initiation site identification. *BMC Bioinformatics* **11**, 119, doi:10.1186/1471-2105-11-119 (2010).

26 Lowe, T. M. & Eddy, S. R. tRNAscan-SE: a program for improved detection of transfer RNA genes in genomic sequence. *Nucleic Acids Res* **25**, 955-964 (1997).

27 Ghai, R., Mehrshad, M., Megumi Mizuno, C. & Rodriguez-Valera, F. Metagenomic recovery of phage genomes of uncultured freshwater actinobacteria. *ISME J* **11**, 304-308, doi:10.1038/ismej.2016.110 (2017).

28 Park, S. J. *et al.* Genomes of two new ammonia-oxidizing archaea enriched from deep marine sediments. *PLoS One* **9**, e96449, doi:10.1371/journal.pone.0096449 (2014).

29 Langille, M. G. & Brinkman, F. S. IslandViewer: an integrated interface for computational identification and visualization of genomic islands. *Bioinformatics* **25**, 664-665, doi:10.1093/bioinformatics/btp030 (2009).

30 Benson, G. Tandem repeats finder: a program to analyze DNA sequences. *Nucleic Acids Res* **27**, 573-580 (1999).

31 Zhou, Y., Liang, Y., Lynch, K. H., Dennis, J. J. & Wishart, D. S. PHAST: a fast phage search tool. *Nucleic Acids Res* **39**, W347-352, doi:10.1093/nar/gkr485 (2011).

32 Wang, Y., Coleman-Derr, D., Chen, G. & Gu, Y. Q. OrthoVenn: a web server for genome wide comparison and annotation of orthologous clusters across multiple species. *Nucleic Acids Res* **43**, W78-84, doi:10.1093/nar/gkv487 (2015).
