# Supplementary material for: Physiological and genomic insights into the lifestyle of arsenite-oxidizing Herminiimonas arsenitoxidans
Source: Sci Rep. 2017 Nov 3;7:15007. doi: 10.1038/s41598-017-15164-4 (PMC5670224; doi:10.1038/s41598-017-15164-4)

**Physiological and genomic insights into the lifestyle of arsenite-oxidizing *Herminiimonas arsenitoxidans***

Hyeon-Woo Koh^1^, Moonsuk Hur^2^, Myung-Suk Kang^2^, Youn-Bong Ku^2^, Rohit Ghai^3^, & Soo-Je Park^1,*^

^1^Department of Biology, Jeju National University, 102 Jejudaehak-ro, Jeju 63243, Republic of Korea

^2^Microorganism Resources Division, National Institute of Biological Resources, 42 Hwangyeong-ro, Incheon 22689, Republic of Korea

^3^Institute of Hydrobiology, Department of Aquatic Microbial Ecology, Biology Center CAS, Na Sadkach 7, České Budějovice 370 05, Czech Republic

***Correspondence:**

Soo-Je Park

Tel.: +82-64-754-3524; Fax: +82-64-756-3541; E-mail: sjpark@jejunu.ac.kr

**Supplementary Material**

**Supplementary Table S1.** The characteristics of the genomic islands of strain AS8 predicted by the IslandViewer.

**Supplementary Table S2.** Insertion sequence prediction of strain AS8.

**Supplementary Table S3.** The intact prophage region identified in the AS8 genome.

**Supplementary Table S4.** The tandem repeats identified in the AS8 genome.

**Supplementary Table S5.** The genes in the AS8 genome involved in carbon, nitrogen, and sulfur metabolism, and respiration.

**Supplementary Table S6.** The genes in the AS8 genome involved in the motility and biofilm formation.

**Supplementary Table S7.** The genes in the AS8 genome involved in arsenic-related stress.

**Supplementary Table S8.** The genes in the AS8 genome involved in osmotic pressure and compatible solutes.

**Supplementary Table S9.** Putative unique CDSs from the AS8 genome determined based on the comparison and annotation of orthologous gene clusters with the *H*. *arsenicoxydans* and *H*. *glaciei* subsp. Marseille genomes.

**Supplementary Figure S1.** Heavy metal concentrations in the contaminated soil samples determined by inductively coupled plasma.

**Supplementary Figure S2.** Two-dimensional thin-layer chromatography analysis of the polar lipids from strain AS8. Abbreviations: diphosphatidylglycerol (DPG); phosphatidylethanolamine (PE); phosphatidylglycerol (PG); phosphatidylserine (PS); unknown aminolipid (AL); and unidentified lipid (L).

**Supplementary Figure S3.** (**a**) The growth (closed circle) of strain AS8 in the absence of arsenite. (**b**) The effect of arsenite concentrations (0, 1, 5, and 10 mM) on the growth rate, within 95% confidence (dotted). (**c**) The resistance of cells to arsenate (0, 1, 5, and 10 to 100 mM, at 10 mM intervals). The cells were cultivated with agitation. The cell density was estimated by optical density (OD) at 600 nm. The error bar represents standard deviations from triplicate experiments.

**Supplementary Figure S4.** (**a**) Circular representation of the AS8 genome. The outermost circle shows rRNA (red) and tRNA (gray) (circle 1), and the gene content is predicted for the reverse and forward strands (circles 2 and 3, respectively). The colors of predicted CDSs are based on the COG functional categories (see the key for the color designations). Circles 4 and 5 show the GC ratio (values higher or lower than the average percentage in the whole genome are shown in yellow and red, respectively) and the GC skew, respectively. (**b**) Distribution of the Clusters of Orthologous Groups (COGs) in the functional classes of the AS8 (black) and *H*. *arsenicoxydans* (gray) genomes. The COG percentage predicted for the two genomes is shown.

**Supplementary Figure S5.** Dot plot representation of the pairwise alignments of the strain AS8 genome with *H*. *arsenicoxydans* (**a**) and *H*. *glaciei* subsp. Marseille (**b**) genomes. The alignments were performed with a six-frame amino acid translation of the genome sequences using the MUMmer 3.23 package. In all plots, a dot indicates a gene compared, with forward and reverse matches shown in red and blue, respectively.

**Supplementary Figure S1.**

**Supplementary Figure S2.**

**
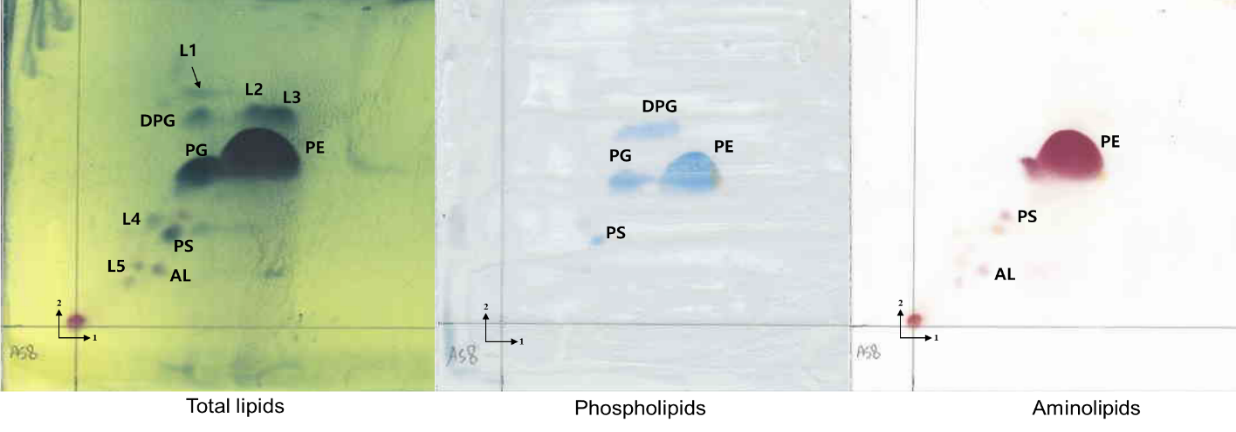
**

**Supplementary Figure S3.**

(a)


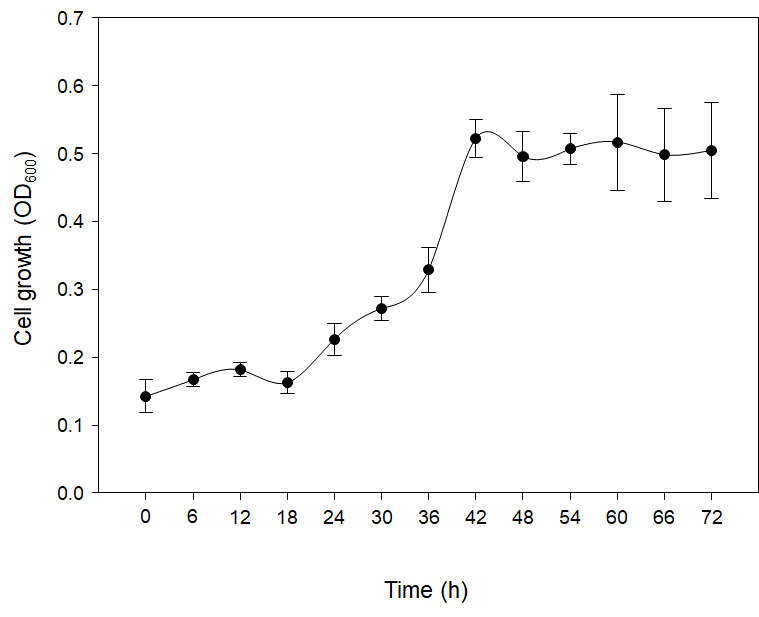


(b)


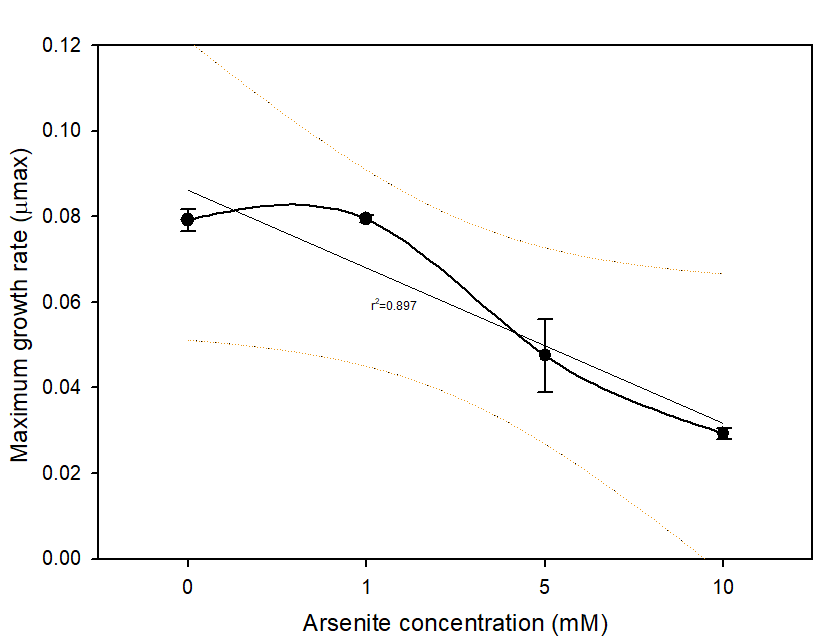


(c)


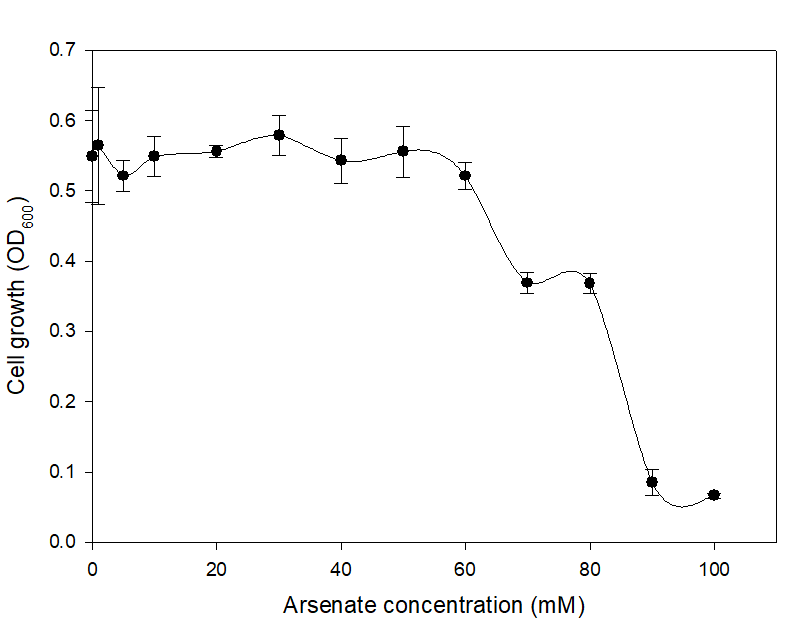


**Supplementary Figure S4.**

(a)


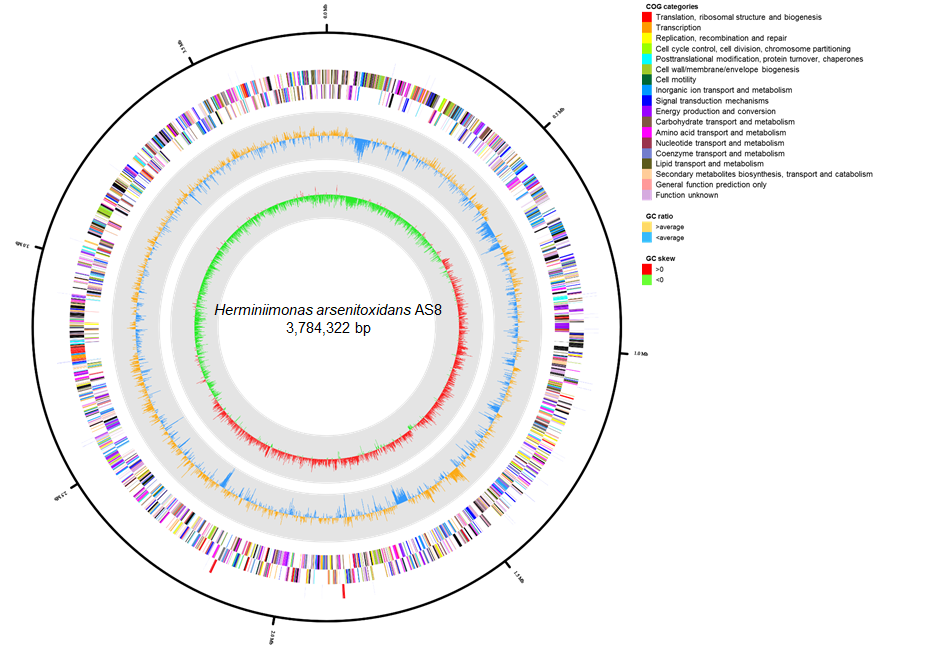


(b)

**Supplementary Figure S5.**

(a)


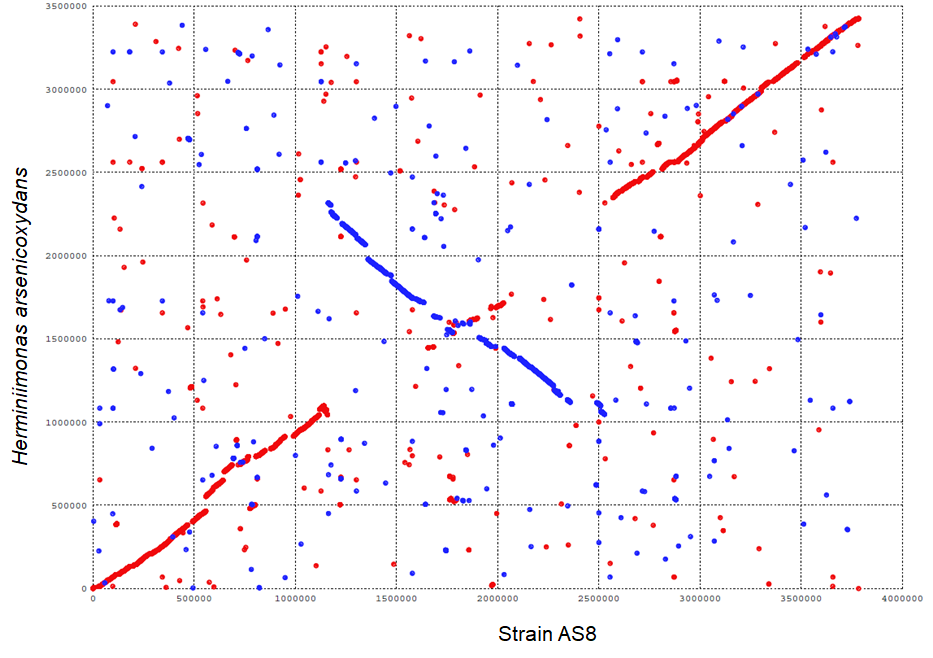


(b)


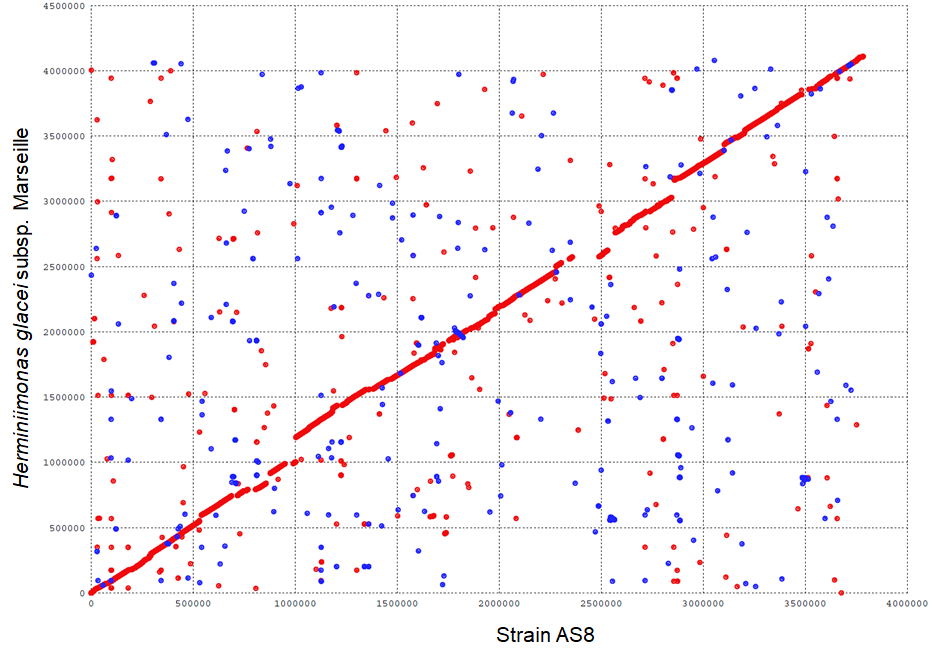

Supplement: Supplementary file 2 — Supplementary Figures [file 41598_2017_15164_MOESM2_ESM.doc]
